# Human palatine tonsil: a new potential tissue source of multipotent mesenchymal progenitor cells

**DOI:** 10.1186/ar2459

**Published:** 2008-07-28

**Authors:** Sasa Janjanin, Farida Djouad, Rabie M Shanti, Dolores Baksh, Kiran Gollapudi, Drago Prgomet, Lars Rackwitz, Arjun S Joshi, Rocky S Tuan

**Affiliations:** 1 Cartilage Biology and Orthopaedics Branch, National Institute of Arthritis and Musculoskeletal and Skin Diseases, National Institutes of Health, Department of Health and Human Services, 9000 Rockville Pike, Bethesda, MD 20892, USA; 2 Department of Otorhinolaryngology, Head & Neck Surgery, Zagreb Clinical Hospital Center, Zagreb University School of Medicine, Kispaticeva 12, 10000 Zagreb, Croatia; 3 Howard Hughes Medical Institute-National Institutes of Health, Research Scholars Program, 1 Cloister Court, Bethesda, MD 20814-1460, USA; 4 Division of Otolaryngology – Head and Neck Surgery, George Washington University, 2150 Pennsylvania Ave. N.W., Washington, DC 20037, USA

## Abstract

**Introduction:**

Mesenchymal progenitor cells (MPCs) are multipotent progenitor cells in adult tissues, for example, bone marrow (BM). Current challenges of clinical application of BM-derived MPCs include donor site morbidity and pain as well as low cell yields associated with an age-related decrease in cell number and differentiation potential, underscoring the need to identify alternative sources of MPCs. Recently, MPC sources have diversified; examples include adipose, placenta, umbilicus, trabecular bone, cartilage, and synovial tissue. In the present work, we report the presence of MPCs in human tonsillar tissue.

**Methods:**

We performed comparative and quantitative analyses of BM-MPCs with a subpopulation of adherent cells isolated from this lymphoid tissue, termed tonsil-derived MPCs (T-MPCs). The expression of surface markers was assessed by fluorescent-activated cell sorting analysis. Differentiation potential of T-MPCs was analyzed histochemically and by reverse transcription-polymerase chain reaction for the expression of lineage-related marker genes. The immunosuppressive properties of MPCs were determined *in vitro *in mixed lymphocyte reactions.

**Results:**

Surface epitope analysis revealed that T-MPCs were negative for CD14, CD31, CD34, and CD45 expression and positive for CD29, CD44, CD90, and CD105 expression, a characteristic phenotype of BM-MPCs. Similar to BM-MPCs, T-MPCs could be induced to undergo adipogenic differentiation and, to a lesser extent, osteogenic and chondrogenic differentiation. T-MPCs did not express class II major histocompatibility (MHC) antigens, and in a similar but less pronounced manner compared with BM-MPCs, T-MPCs were immunosuppressive, inhibiting the proliferation of T cells stimulated by allogeneic T cells or by non-specific mitogenic stimuli via an indoleamine 2,3-dioxygenase-dependent mechanism.

**Conclusion:**

Human palatine T-MPCs represent a new source of progenitor cells, potentially applicable for cell-based therapies.

## Introduction

Mesenchymal progenitor cells (MPCs), originally discovered in bone marrow (BM) stroma, support hematopoiesis and can differentiate along multiple mesenchymal lineages, including osteoblasts, chondrocytes, adipocytes, and myocytes [1-3]. Due to their differentiation capacities, MPCs have emerged as a promising tool for therapeutic applications in tissue engineering and cell and gene therapy. Animal studies have shown that MPC implantation can repair critical bone fracture in a rat model of femoral segmental defect [4] and that, after systemic injection, MPCs localize to the site of experimentally induced fractures [5]. Pilot clinical studies have demonstrated the feasibility of allogeneic BM transplantation in the treatment of osteogenesis imperfecta. BM-derived MPCs (BM-MPCs) engrafted and generated donor-derived osteoblasts that improved the clinical signs associated with the disease and enhanced total body weight [6]. Besides their multilineage potential, MPCs display immunoregulatory properties that have prompted consideration of their use in BM transplantation. Indeed, a recent study reports the successful use of MPCs to treat severe grade IV acute graft-versus-host disease in one patient after allogeneic hematopoietic stem cell transplantation [7]. Although the exact immunosuppressive mechanisms are unknown, the capacity of MPCs to suppress T-cell proliferation stimulated by allogeneic lymphocytes, dendritic cells, and phytohemaglutinin (PHA) is well documented [8]. Mechanisms involving cell contact. [9] as well as soluble factors [10,11] have been proposed, particularly the involvement of interferon-gamma (IFN-γ) via its induction of indoleamine 2,3-dioxygenase (IDO), an enzyme involved in the catabolism of tryptophan, an essential amino acid required for protein synthesis and T-cell proliferation [12,13].

At present, BM is considered the most accessible source of adult MPCs. However, BM-MPC derivation has complications, including pain, donor site morbidity, and low cell yields upon harvest. Furthermore, the number of BM-MPCs and their proliferation rate and differentiating potential have been shown to decrease with donor age. [14]. Given that MPCs undergo a decline in their differentiation and expansion capacity with physiological aging, identification of potential sources of MPCs easily accessible from young donors is currently of main interest for cell-based therapy. [15]. The search for alternative sources of MPCs is thus of significant value. To date, MPCs have been isolated from a number of adult tissues, including trabecular bone [16], fat [17,18], synovium. [19,20], skin [21], thymus [22], periodontal ligament. [23] as well as prenatal and perinatal tissues such as umbilical cord blood. [24], umbilical cord [25], and placenta. [26].

This study explores the possibility of identifying and isolating MPCs from human palatine tonsils. Tonsillar epithelium is derived from the second pharyngeal pouch (of endodermal origin) and during fetal development is invaded by lymphoid tissue (of mesodermal origin). Therefore, embryologically, tonsils could be a source of MPCs. Because of the prevalence of tonsillectomy procedure, tonsils are easily accessible, particularly from young donors and, if necessary, tonsillar biopsy can be easily obtained without major complications under local anesthesia. Our results show that MPCs exist in the stroma of palatine tonsils and can be isolated and expanded in culture. These tonsil-derived MPCs (T-MPCs) show multipotent differentiation properties and share similar immunosuppressive characteristics as BM-MPCs in mixed lymphocyte reaction (MLR). The immunosuppressive activity is significant and dose-dependent, though at a lower level than that of BM-MPCs. The difference in immuosuppressive activity correlates with the level of cell surface IFN-γ receptor (IFN-γR) as well as the differential ability of IFN-γ to stimulate IDO activity by T-MPCs compared with BM-MPCs.

## Materials and methods

### T-MPC and BM-MPC isolation and culture

With institutional review board approval (George Washington University, Washington, DC, USA), tonsils were obtained after informed consent from patients (4 to 15 years old) undergoing tonsillectomy as a result of recurrent episodes of acute tonsillitis. The tissue was minced and digested in RPMI medium (Gibco-BRL, now part of Invitrogen Corporation, Carlsbad, CA, USA) containing 210 U/mL collagenase type I (Invitrogen Corporation) and 90 KU/mL DNase (Sigma-Aldrich, St. Louis, MO, USA) for 30 minutes at 37°C. Following filtration through a wire mesh, the cells were washed twice in 20% normal human serum (NHS)-RPMI and once with 10% NHS-RPMI. Mononuclear cells were obtained by Ficoll-Paque (Amersham, now part of GE Healthcare, Little Chalfont, Buckinghamshire, UK) density gradient centrifugation of digested tonsil tissue. Cells were plated after 24 to 48 hours in T-150 cm^2 ^tissue culture flasks (Corning Incorporated, Corning, NY, USA), and non-adherent cells were washed away with expansion medium consisting of Dulbecco's modified Eagle's medium (DMEM) (Invitrogen Corporation) with 10% fetal bovine serum (FBS) from selected lots (HyClone, Logan, UT, USA) and antibiotics (50 μg/mL streptomycin and 50 IU/mL penicillin; Invitrogen Corporation).

For BM-MPCs, BM was obtained after informed consent from patients (39 to 58 years old) undergoing lower extremity reconstructive surgery with institutional review board approval (University of Washington, Seattle, WA, USA, and George Washington University) and was processed by direct plating as described previously. [27]. BM aspirates were plated overnight in T-150 cm^2 ^culture flasks in the same expansion medium as T-MPCs, and adherent cells were obtained similarly. Both T-MPCs and BM-MPCs were culture-expanded in basal medium at 37°C and 5% CO_2 _using T-150 Triple Flask (Nunc, Roskilde, Denmark), and medium changes were done twice weekly.

### Cell proliferation, limiting dilution assays, and colony-forming unit-fibroblast assays

To estimate cell proliferation, cultures of T-MPCs and BM-MPCs plated at 1 × 10^4 ^cells per square centimeter in 12-well plates in basal medium were analyzed on days 3, 4, 7, 10, 12, and 14, using MTS (methanethiosulfonate) assay according to the protocol of the manufacturer (Promega Corporation, Madison, WI, USA). T-MPCs plated into six-well plates in serial dilutions (1 × 10^7^, 1 × 10^6^, 1 × 10^5^, and 1 × 10^4 ^cells per well, in triplicate) were cultured in expansion medium for 2 weeks, fixed with 10% formalin, and stained with Giemsa (Sigma-Aldrich), and colonies of fibroblast-like cells were identified and counted based on the methods described by Castro-Malaspina and colleagues. [28]. Colony-forming unit-fibroblast (CFU-F) potential, the average number of cells required to produce one colony, was determined by plating aliquots (1,000 cells per square centimeter) of T-MPCs in expansion medium for 14 days and was analyzed as described previously [29].

### Cell surface epitope profiling

For immunofluorescence, undifferentiated cells were washed twice in phosphate-buffered saline (PBS) (Invitrogen Corporation), fixed with 4% paraformaldehyde in PBS (FD NeuroTechnologies, Inc., Ellicott City, MD, USA) for 15 minutes, and then permeabilized in a PBS solution containing 300 mM sucrose, 3 mM MgCl_2_, and 0.5% (vol/vol) Triton X-100 (Bio-Rad Laboratories, Inc., Hercules, CA, USA) for 5 minutes at 4°C. Cells were stained for cell surface markers (negative markers: CD14, CD34, and CD45; positive markers: CD29, CD44, and CD105) using specific mouse monoclonal antibodies (all obtained from BD Biosciences, San Jose, CA, USA) at 0.5 ng/μL for 2 hours. Secondary immunostaining was done with Alexa Fluor 488 conjugated goat anti-mouse immunoglobulin (diluted at 1:400) (Molecular Probes Inc., now part of Invitrogen Corporation) for 1 hour. Nuclear counterstaining was done with 4',6-diamidino-2-phenylindole dihydrochloride (DAPI) (Invitrogen Corporation) for 5 minutes at 12 μg/30 mL PBS. Immunostained cultures were mounted with Fluoromount-G (Southern Biotech, Birmingham, AL, USA) and observed using confocal laser scanning microscopy (Zeiss LSM 510; Carl Zeiss, Jena, Germany). For flow cytometry, T-MPCs (>1 × 10^5 ^cells) were washed and resuspended in PBS + 0.1% FBS (PF) containing saturating concentrations (1:100 dilution) of the following conjugated mouse IgG_1,κ _anti-human monoclonal antibodies (BD Biosciences): HLA-A, B, C-phycoerythrin (PE) (MHC I), HLA-DR, DP, DQ-fluorescein isothiocyanate (FITC) (MHC II), CD45-FITC, CD14-PE, CD31-PE, CD34-PE, CD73-PE, CD90-FITC, CD105-PE as well as IFN-γR1-PE (R&D Systems, Inc., Minneapolis, MN, USA) for 1 hour at 4°C. Cell suspensions were washed twice and resuspended in PF for analysis on a flow cytometer (FACSCalibur; BD Biosciences) using the CellQuest ProTM software (BD Biosciences).

### *In vitro *differentiation

T-MPCs and BM-MPCs were induced to undergo adipogenic, osteogenic, and chondrogenic differentiation as described previously. [27]. For adipogenic differentiation, cells were seeded into six-well tissue culture plates at a density of 20,000 cells per square centimeter and treated for 3 weeks with adipogenic medium, consisting of DMEM with 10% FBS, and supplemented with 0.5 mM 3-isobutyl-1-methylxanthine (IBMX), 1 μg/mL insulin, and 1 μM dexamethasone (all from Sigma-Aldrich). For osteogenic differentiation, cells were seeded into six-well plates (Corning Incorporated) at a density of 20,000 cells per square centimeter and treated for 3 weeks with osteogenic medium, consisting of DMEM with 10% FBS, and supplemented with 10 mM β-glycerolphosphate, 10 nM dexamethasone, 50 μg/mL ascorbic acid-2-phosphate, and 10 nM 1,25 dihydroxyvitamin D_3 _(Biomol International L.P., Plymouth Meeting, PA, USA). To induce chondrogenic differentiation, 96-microwell polypropylene plates (Nunc) were seeded with 300,000 cells per well, and cell pellets formed by centrifugation at 1,100 rpm for 6 minutes. The pellet cultures were treated for 3 weeks with chondrogenic medium, consisting of high-glucose DMEM supplemented with 100 nM dexamethasone, 50 μg/mL ascorbic acid-2-phosphate, 100 μg/mL sodium pyruvate, 40 μg/mL L-proline, 10 ng/mL recombinant human transforming growth factor-β3 (R&D Systems, Inc.), and 50 mg/mL insulin-transferrin-selenium (ITS)-premix stock (BD Biosciences).

### Histology and histochemistry

#### Oil red O staining

Three-week adipogenic cultures of MPCs were rinsed twice with PBS, fixed in 4% buffered paraformaldehyde, stained with Oil red O (Sigma-Aldrich) for 5 minutes at room temperature, and counterstained with Harris-hematoxylin solution (Sigma-Aldrich) to visualize lipid droplets.

#### Alizarin red S staining

MPCs cultured for 3 weeks in osteogenic medium were fixed with 60% isopropyl alcohol and stained for 3 minutes with 2% (wt/vol) Alizarin red S (Rowley Biochemical Inc., Danvers, MA, USA) at pH 4.2 to detect mineralization.

#### Alcian blue staining

Chondrogenic cell pellets were fixed in 4% buffered paraformaldehyde, rinsed with PBS, serially dehydrated, paraffin-embedded, and sectioned at 10-μm thickness for histological staining with Alcian blue (pH 1.0) for sulfated proteoglycans.

### Total RNA isolation and real-time reverse transcription-polymerase chain reaction

Total cellular RNA samples extracted from day 21 monolayer and pellet cultures using Trizol Reagent (Invitrogen Corporation) were reverse-transcribed using random hexamers. Real-time polymerase chain reactions were performed using 10 ng of cDNA and SYBR Green mix (Bio-Rad Laboratories, Inc.). Gene-specific primers (forward/reverse) were designed based on GenBank cDNA sequences and are listed in Table 1: (a) adipogenesis genes: lipoprotein lipase (*LPL*) and peroxisome proliferator-activated receptor-gamma (*PPARγ*), (b) osteogenesis genes: alkaline phosphatase (*ALP*) and osteocalcin (*OC*), and (c) chondrogenesis genes: collagen type II α1 (*COL2*) and aggrecan (*AGN*). Specific transcript levels were normalized by comparison to that of the housekeeping gene, glyceraldehyde-3-phosphate dehydrogenase (*GAPDH*). Expression levels were presented as the fold increase over that of GAPDH, using the formula 2^(ΔCt)^, where ΔCt = Ct of target gene – Ct of GAPDH.

**Table 1:** Reverse transcription-polymerase chain reaction primers for differentiation-specific gene expression analysis

Genes	Primer sequences (5'-3')	Position, base pairs	Predicted size, base pairs
Housekeeping gene			
*GAPDH*	Sense: GGACTCATGACCACAGTCCATGCC	619–770	152
	Antisense: TCAGGGATGACCTTGCCCACA		
Bone-specific genes			
*ALP*	Sense: TGGAGCTTCAGAAGCTCAACACCA	379–832	454
	Antisense: ATCTCGTTGTCTGAGTACCAGTCC		
*OC*	Sense: ATGAGAGCCCTCACACTCCTC	19–312	294
	Antisense: GCCGTAGAAGCGCCGATAGGC		
Adipose-specific genes			
*LPL*	Sense: GAGATTTCTCTGTATGGCACC	1,457–1,732	276
	Antisense: CTGCAAATGAGACACTTTCTC		
*PPARγ*	Sense: TGAATGTGAAGCCCATTGAA	1,476–1,636	161
	Antisense: CTGCAGTAGCTGCACGTGTT		
Cartilage-specific genes			
*AGN*	Sense: TGCGGGTCAACAGTGCCTATC	655–836	182
	Antisense: CACGATGCCTTTCACCACGAC		
*COL2A1*	Sense: GGAAACTTTGCTGCCCAGATG	710–876	167
	Antisense: TCACCAGGTTCACCAGGATTGC		

AGN, aggrecan; ALP, alkaline phosphatase; COL2A1, collagen type II α1; GAPDH, glyceraldehyde 3-phosphate dehydrogenase; LPL, lipoprotein lipase; OC, osteocalcin; PPARγ, peroxisome proliferator-activated receptor-gamma.

### Primary mixed lymphocyte reaction

Peripheral blood from healthy human donors was collected into heparinized containers (BD Biosciences), and peripheral blood mononuclear cells (PBMCs) were isolated by Ficoll-Hypaque density gradient centrifugation. Mouse splenocytes were isolated in 10 mL of RPMI 1640 medium (Invitrogen Corporation) as described previously. [30]. Responder human PBMCs or splenocytes from CD-1 mice and stimulator human PBMCs or splenocytes from A/J mice were resuspended in RPMI 1640 medium containing 10% FBS, 2 mM glutamine, 100 U/mL penicillin and 100 μg/mL streptomycin, 0.1 mM non-essential amino acids, 1 mM sodium pyruvate, 20 mM HEPES, and 50 μM 2-mercaptoethanol (Invitrogen Corporation). Splenocytes were seeded in triplicates at 1 × 10^5 ^cells/100 μL per well in 96-well round-bottom plates (BD Biosciences). PHA was used at 5 μg/mL as a positive control mitogen to induce T-cell proliferation. MPCs (5 × 10^4 ^cells unless stated otherwise) were added to obtain a final volume of 300 μL. After 3 days of incubation, 1 μCi/well [^3^H]-thymidine (GE Healthcare) was added overnight and radioactivity incorporation was determined by liquid scintillation counting. All experiments were performed in triplicates and repeated at least twice.

### Indoleamine 2,3-dioxygenase activity assay

Cells were stimulated with IFN-γ (100 ng/mL) for 72 hours in DMEM supplemented with L-tryptophan (100 μg/mL). In some experiments, a neutralizing antibody (anti-IFN-γR1, 1.5 μg/mL; R&D Systems, Inc.) was added to the cultures. IDO enzyme activity in the culture supernatant was measured spectrophotometrically based on tryptophan-to-kynurenine conversion, as described previously. [20].

### Interferon-gamma assay

IFN-γ in culture supernatants was quantified using a commercially available enzyme-linked immunosorbent assay kit (R&D Systems, Inc.) according to the manufacturer's protocol.

### Statistics

Data from the proliferation, real-time reverse transcription-polymerase chain reaction (RT-PCR), and IDO activity assays were analyzed statistically using the Student *t *test, with statistical significance set at a *P *value of less than 0.05.

## Results

### Cell viability, proliferation, and clonogenicity

**Figure 1:**
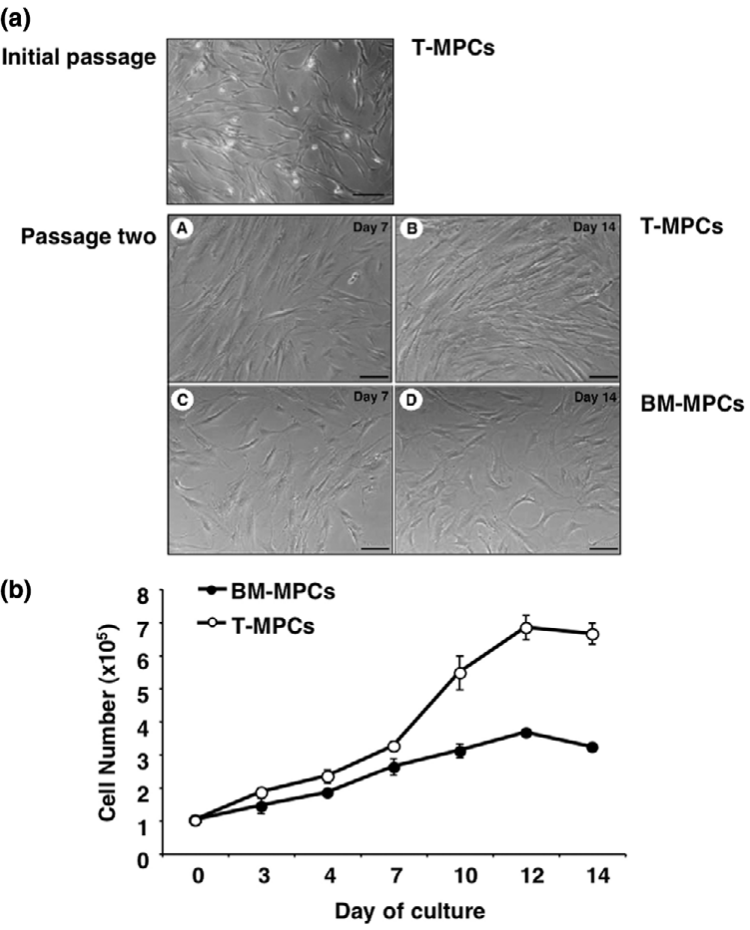
Characteristics of tonsil-derived mesenchymal progenitor cells (T-MPCs). **(a) **Morphology of T-MPCs at initial passage. Two different types of cell morphologies are apparent under phase-contrast microscopy: a fibroblast-like spindle-shaped morphology and a round morphology with large nuclei (monocytic contamination). The morphology of passage 2 bone marrow-derived mesenchymal progenitor cells (BM-MPCs) and T-MPCs in culture is shown. Cultures were observed at day 7 and day 14. T-MPCs **(A, B)**; BM-MPCs **(C, D)**. The two cell types display similar fibroblastic morphologies. Bars = 20 μm. **(b) **Proliferation kinetics of T-MPCs and BM-MPCs analyzed by MTS (methanethiosulfonate) assay. T-MPCs and BM-MPCs were plated at the same initial density (1 × 10^5 ^cells per plate). A difference in the proliferation rates of T-MPCs and BM-MPCs is observed, with T-MPCs proliferating at a faster rate than BM-MPCs throughout the assay period. Values are mean ± standard deviation (n = 9).

The cell yield from each tonsil ranged from 1 to 5 × 10^9^, with the majority being non-adherent and likely of hematopoietic origin. After multiple buffer washes and subsequent medium changes, approximately 0.1% to 1% of the isolated cells were found to be adherent. Cell colonies from processed tonsillar specimens began to appear approximately 5 to 10 days after initial plating. Three different cell morphologies were generally observed: (a) fibroblast-like spindle-shaped morphology, (b) round morphology and large nuclei (monocytic contamination), and (c) very small, epitheloid cells with polygonal morphology (Figure 1a). After trypsinization at each passage, the round cell population remained attached to the flasks, and was no longer detected by passage 2, confirmed subsequently by negative expression of CD14, a myelomonocytic marker. The small epitheloid cells rapidly disappeared from the culture as early as passage 1. At later passages, T-MPCs were homogeneously fibroblast-like, with extended cytoplasmic processes. Morphologically, these cells were indistinguishable from BM-MPCs at similar passage numbers (Figure 1a). In general, T-MPCs were somewhat smaller than BM-MPCs, with a cell yield of 7 to 10 × 10^6 ^cells per 80% confluent Nunc triple flask compared with 4 to 8 × 10^6 ^BM-MPCs. The proliferation profiles of T-MPCs and BM-MPCs were distinctly different (Figure 1b). Plated at the same initial cell number, T-MPCs proliferated at a faster rate compared with BM-MPCs throughout the assay period. The population doubling times for T-MPCs and BM-MPCs were calculated to be 37.1 ± 3.4 hours and 58.2 ± 2.3 hours, respectively. By day 14, T-MPCs and BM-MPCs underwent 2.70 ± 0.13 and 1.69 ± 0.06 population doublings, respectively. Upon plating at limiting dilution under CFU-F assay conditions, the initial isolates of T-MPCs showed a linear relationship between colony number and cell number, suggesting that one T-MPC was limiting for CFU-F. The CFU-F frequency in the tonsil digest was determined to be approximately 1 in 6,000 cells plated.

### Immunofluorescence and flow cytometric analyses

**Figure 2:**
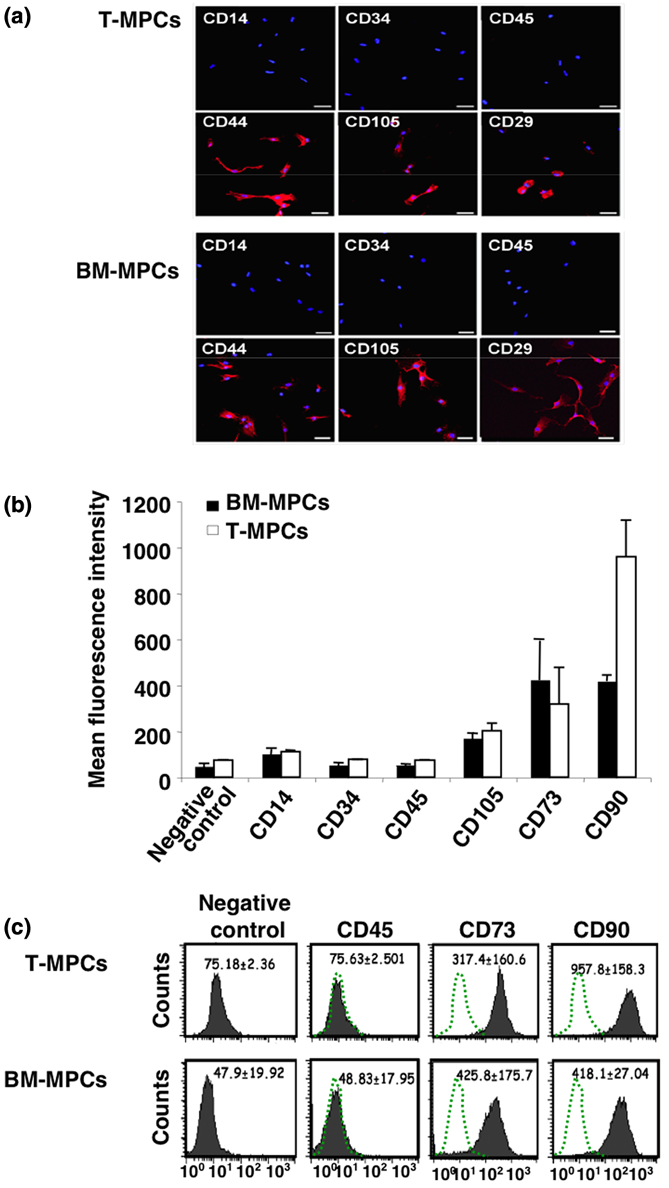
Surface epitope profile of tonsil-derived mesenchymal progenitor cells (T-MPCs). **(a) **Immunofluorescence analysis of cell surface epitope profiles of T-MPCs and bone marrow-derived mesenchymal progenitor cells (BM-MPCs). T-MPCs are shown in the top two rows of panels, and BM-MPCs are shown in the bottom two rows of panels. Epitopes were detected using fluorescently labeled secondary antibodies (red). Nuclei were stained with DAPI (blue). Both cell populations were negative for CD14, CD34, and CD45 and positive for CD29, CD44, and CD105. Bars = 20 μm. **(b) **Flow cytometric analysis of T-MPCs and BM-MPCs. CD14, CD34, CD45, CD105, CD73, and CD90 were detected by fluorescently conjugated antibodies. The level of expression of each epitope is expressed as the mean fluorescence intensity ± standard deviation (n = 3). **(c) **Representative flow cytometry histogram. Control represents fluorescence due to the isotypic control. DAPI, 4',6-diamidino-2-phenylindole dihydrochloride.

T-MPCs and BM-MPCs expressed similar surface epitope profiles (that is, positive/negative for the same cell markers). Based on immunofluorescence staining, T-MPCs and BM-MPCs were both positive for CD29, CD44, and CD105 and negative for CD14, CD34, and CD45 (Figure 2a). Similar to BM-MPCs, T-MPCs were positive for MHC class I molecules and negative for MHC class II molecules in basal culture conditions (data not shown). Flow cytometric analysis of T-MPCs confirmed that T-MPCs were non-hematopoietic cells based on their lack of CD45 and unlikely to be of endothelial origin (negative for CD31; data not shown). Importantly, T-MPCs exhibited a similar cell surface epitope phenotype as BM-MPCs, specifically expressing CD105, CD73, and CD90 (Figures 2b and 2c). Fluorescence intensities for these markers were not statistically different between the two cell populations, suggesting a similar level of expression in both cell populations, except for CD90 (*P *= 0.022), which was higher in T-MPCs.

### Multilineage differentiation potential

#### Adipogenesis

**Figure 3:**
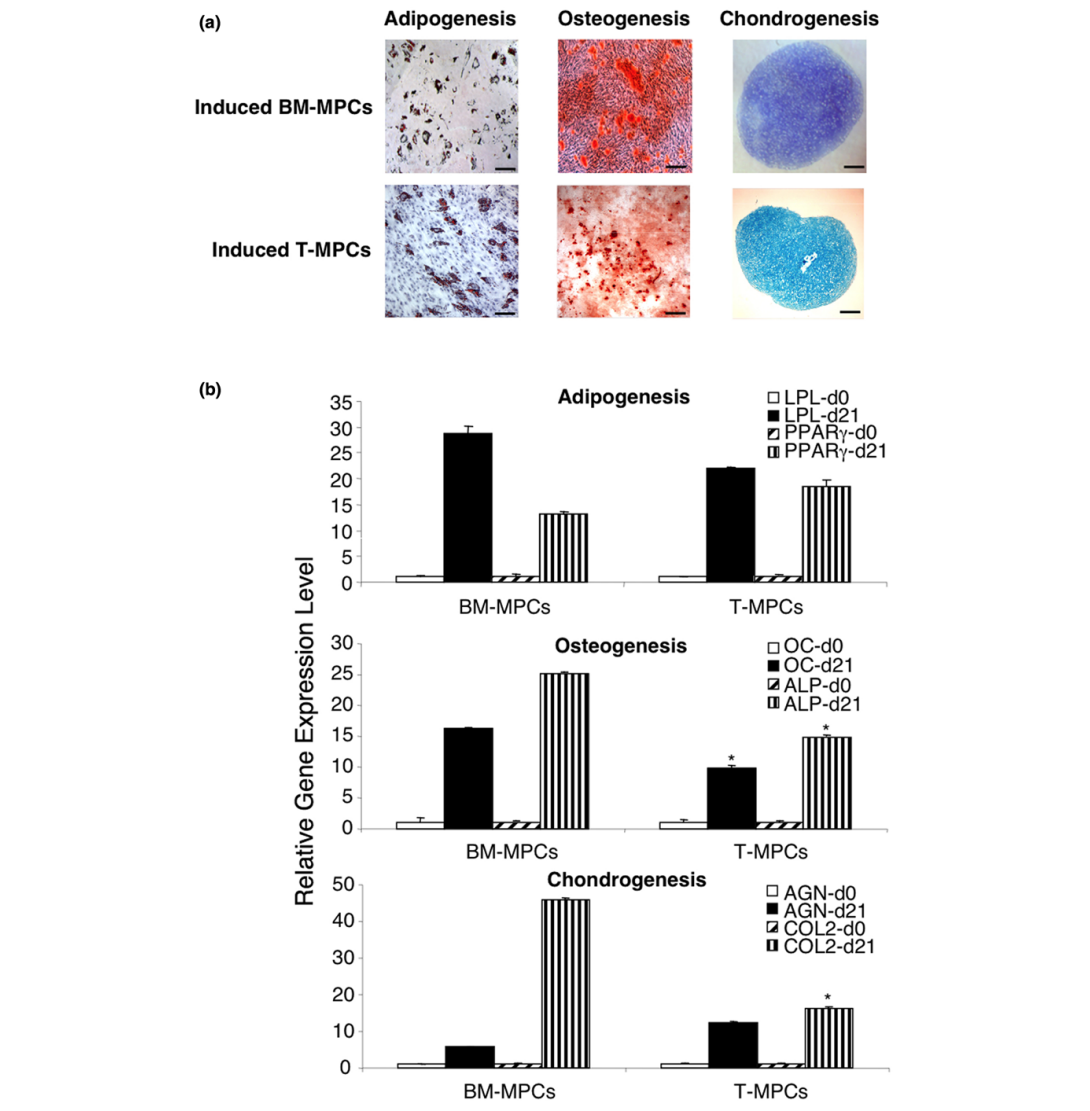
Histological and real-time reverse transcription-polymerase chain reaction analysis of adipogenic, osteogenic, and chondrogenic differentiation of tonsil-derived mesenchymal progenitor cells (T-MPCs). **(a) **Histology. Induced bone marrow-derived mesenchymal progenitor cell (BM-MPC) cultures are shown in the top panels; induced T-MPC cultures are shown in the bottom panels. In adipogenesis, the formation of lipid droplets is visualized by staining with Oil red O (bars = 40 μm); in osteogenesis, the matrix mineralization is shown by Alizarin red staining (bars = 40 μm); and in chondrogenesis, the accumulation of sulfated glycosaminoglycan-rich matrix is detected by Alcian blue staining (bars = 300 μm). **(b) **Gene expression analysis of adipogenic, osteogenic, and chondrogenic differentiation of T-MPCs in comparison with BM-MPCs. Adipogenesis genes are lipoprotein lipase (*LPL*) and proliferator-activated receptor-gamma (*PPARγ*), osteogenesis genes are osteocalcin (*OC*) and alkaline phosphatase (*ALP*), and chondrogenesis genes are aggrecan (*AGN*) and collagen type II α1 (*COL2*). Gene expression analysis was done at the beginning of culture (d0) and at 3 weeks (d21). Expression levels were normalized on the basis of glyceraldehyde 3-phosphate dehydrogenase (*GAPDH*) expression, and the results are reported as ratios of the marker gene versus *GAPDH *using the formula 2^ΔCT ^(× 100). Values are mean ± standard deviation (n = 2). *P < 0.05 versus BM-MPCs.

Passage 2–5 T-MPCs were treated with adipogenic supplements, with controls including T-MPCs and BM-MPCs of the same passage maintained and cultured in expansion medium, and BM-MPCs cultured in adipogenic medium. Morphological changes in BM-MPCs and T-MPCs and the formation of cytoplasmic lipid droplets were noticeable as early as 1 week of adipogenic induction, as visualized by Oil red O staining (Figure 3a). mRNA expression of *LPL *and *PPARγ *was detected by quantitative RT-PCR (Table 1) after 21 days of induction and revealed a similar expression level of the two markers in T-MPCs and BM-MPCs (Figure 3b). Interestingly, expression of adipogenic markers was significantly stronger in higher passages of T-MPCs (P4 and P5) than in lower passages (P2 and P3) (data not shown).

#### Osteogenesis

Upon osteogenic induction, the morphology of both T-MPCs and BM-MPCs changed from spindle-shaped to flattened and spread. Quantitative RT-PCR analysis showed lower levels of *OC *and *ALP *mRNA in T-MPCs compared with BM-MPCs (Figure 3b). Osteoblastic phenotype was also detected based on positive staining for ALP (not shown) and Alizarin red S (Figure 3a).

#### Chondrogenesis

The chondrogenic potential of BM-MPCs and T-MPCs was evaluated in high-density pellet cultures maintained in serum-free chondrogenic medium. After 3 weeks of culture, matrix sulfated proteoglycan accumulation in chondrogenic cultures was detectable by Alcian blue staining (Figure 3a). Quantitative RT-PCR analysis revealed a significant increase of *AGN *and *COL2 *expression in both T-MPCs and BM-MPCs, although the increase in *COL2 *expression was significantly lower in T-MPCs pellets compared with BM-MPCs (Figure 3b).

### Inhibition of proliferation of alloreactive T cells

**Figure 4:**
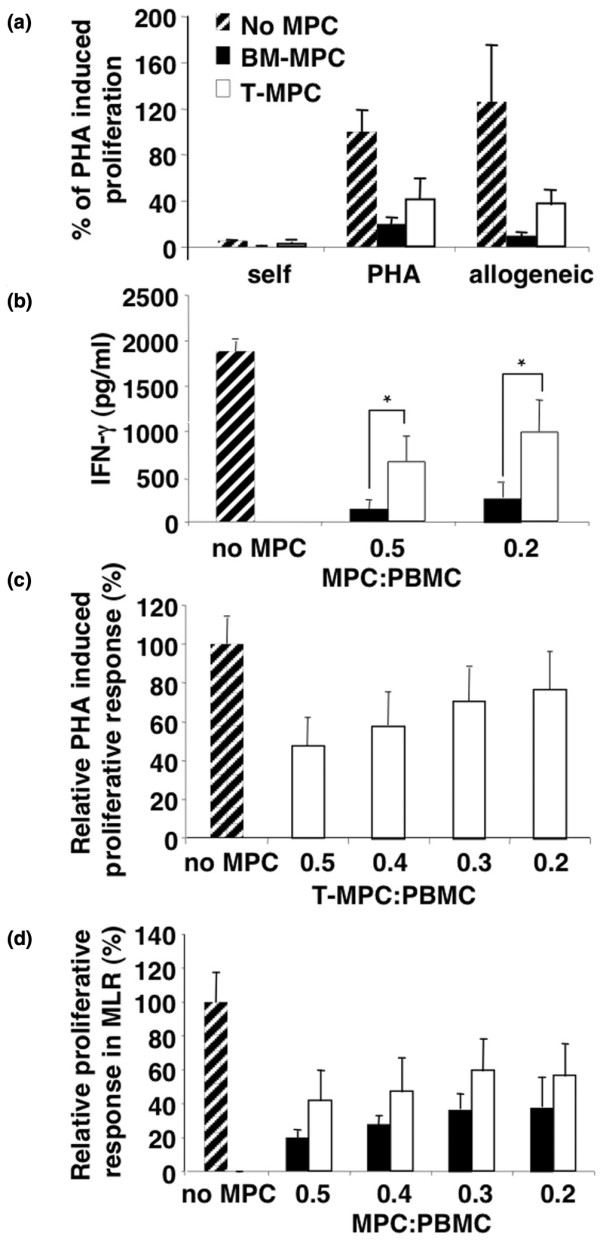
Tonsil-derived mesenchymal progenitor cells (T-MPCs) inhibit allogeneic as well as phytohemaglutinin (PHA)-induced proliferative response in a dose-dependent manner regardless of the species of T cells. Responding peripheral blood mononuclear cells (PBMCs) (10^5 ^cells) were incubated for 3 days with either 5 μg/mL PHA or allogeneic stimulating PBMCs (1 × 10^5 ^cells) with or without bone marrow-derived mesenchymal progenitor cells (BM-MPCs) or T-MPCs (5 × 10^4 ^or varying ratios). **(a) **Cell proliferation based on [^3^H]-thymidine incorporation. BM-MPCs and T-MPCs inhibit the T-cell receptor-independent (PHA) and -dependent (allogeneic) T-cell proliferative response. The proliferative response (counts per minute per culture) of PHA-induced T-cell proliferation was assigned the value of 100%. All values are mean ± standard deviation (SD) of triplicates. **(b) **Interferon-gamma (IFN-γ) levels determined by enzyme-linked immunosorbent assay. IFN-γ levels in supernatants obtained from a 3-day proliferative assay using PBMCs stimulated with 5 μg/mL PHA with or without BM-MPCs and T-MPCs at the indicated ratios. Values are mean ± SD (n = 3) and *, *P *< 0.05 versus BM-MPCs. **(c) **Dose-dependent inhibitory effect of T-MPCs on PHA-induced T-cell proliferation. T-MPCs exhibit a dose-dependent inhibition of PHA-induced T-cell proliferation. Results (mean ± SD, n = 3) are expressed as the percentage of T-cell proliferation obtained in the absence of T-MPCs. **(d) **Dose-dependent inhibitory effect of T-MPCs and BM-MPCs on T-cell proliferative response induced by xenogeneic murine splenocytes in a mixed lymphocyte reaction (MLR). Results (mean ± SD, n = 3) are expressed as the percentage of the responder-stimulator pair response in the absence of MPCs. T-MPCs and BM-MPCs inhibit the T-cell proliferative response in a dose-dependent manner.

The MLR was used to test the immunosuppressive properties of T-MPCs and BM-MPCs. Initially, using human PBMCs from healthy donors as responding cells and PHA as a mitogen, the addition of BM-MPCs and T-MPCs both inhibited the PHA-induced proliferative response of PBMCs (Figure 4a). In the MLR, BM-MPCs and T-MPCs were also seen to suppress the proliferation of responder PBMCs, elicited by allogeneic PBMCs (Figure 4a). In parallel, the level of IFNγ, which reflected T-cell proliferation, showed a decrease in the MLR proportionally in the presence of MPCs isolated from both tissues, but to a significantly lower extent with T-MPCs, supporting a less potent suppressive activity of these cells (Figure 4b). Proliferation suppression by T-MPCs was dose-dependent and was partially reversed at a T-MPC-to-responder cell ratio of 1:5, suggesting a potent effector mechanism (Figure 4c). Indeed, similar to BM-MPCs, T-MPC suppression of T-cell proliferation was dose-dependent for PHA stimulation as well as in an MLR. However, the immunomodulatory activity of T-MPCs was significantly less pronounced than that of BM-MPCs. The immunosuppressive activities of human BM-MPCs and T-MPCs also crossed the species barrier. When CD-1 murine splenocytes were stimulated with allogeneic A/J splenocytes, dose-dependent inhibition of the proliferative response was seen when xenogeneic human BM-MPCs and T-MPCs were added (Figure 4d).

### Involvement of interferon-gamma and indoleamine 2,3-dioxygenase in MPC-mediated immunosuppression

**Figure 5:**
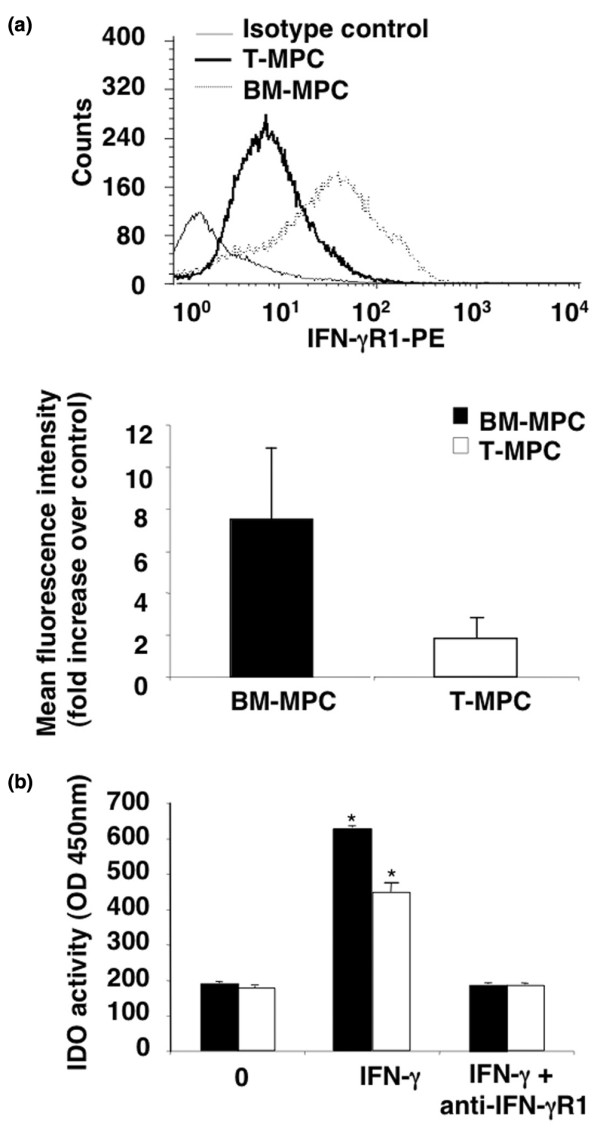
Interferon-gamma receptor-1 (IFN-γR1) expression and indoleamine 2,3-dioxygenase (IDO) activity in bone marrow-derived mesenchymal progenitor cells (BM-MPCs) and tonsil-derived mesenchymal progenitor cells (T-MPCs). **(a) **Basal expression level of IFN-γR1. BM-MPCs (n = 4) and T-MPCs (n = 5) were analyzed by flow cytometry. **(b) **IDO activity. BM-MPCs (n = 3) and T-MPCs (n = 4) were cultured in the absence or presence of IFN-γ (100 ng/mL) for 72 hours. Neutralizing anti-IFN-γR1 antibody (1.5 μg/mL) was also tested on cells cultured with IFN-γ. IDO activity was assayed as described in Materials and methods. No IDO activity was detected in the absence of IFN-γ. The differential induction of IDO activity by T-MPCs and BM-MPCs stimulated by IFN-γ is reversed in the presence of neutralizing anti-IFN-γR1 antibody. OD, optical density; PE, phycoerythrin. *, *P *< 0.05 versus control.

MPC immunosuppression was recently shown to require IFN-γ (produced by T cells and natural killer [NK] cells) to stimulate IDO production by MPCs, which in turn inhibits the proliferation of activated T or NK cells. [13]. Also, treatment with neutralizing anti-IFN-γR antibody completely abrogated the immunosuppressive effect of MPCs. Analysis of BM-MPCs and T-MPCs showed that both cell types expressed IFN-γR1, with a substantially higher level in the former (Figure 5a). Incubation with IFN-γ resulted in induction of IDO activity in both cell populations, with a lower level in T-MPCs (Figure 5b). Furthermore, the IFN-γ-induced IDO activity was completely suppressed by neutralizing anti-IFN-γR1 antibody. These findings thus strongly suggested that the immunsuppressive activities of both BM-MPCs and T-MPCs were mediated via IDO activity, with BM-MPCs being more active. This differential inhibitory ability correlated with a reduced capacity of T-MPCs to decrease IFN-γ secretion as well as to induce their IDO activity as compared with BM-MPCs (Figure 5b).

## Discussion

The purpose of this study was twofold: (a) to assess the existence of MPCs in human palatine tonsils and to characterize their phenotype, and (b) to determine and compare the differentiation potential and immunomodulatory activity of these cells (T-MPCs) to those of the well-characterized MPCs isolated from BM (BM-MPCs). The results showed that human palatine tonsils contained a multipotent MPC population. By means of standard procedure, T-MPCs can be successfully isolated and expanded *in vitro*. The initial cell population (post tonsil digest) was contaminated with non-fibroblastoid tissue culture adherent cell types; specifically, monocytes remained attached to the polystyrene flasks even after extensive trypsinization, while epitheloid cells did not survive under the expansion medium conditions used to grow BM-MPCs. Only the fibroblastoid cell population remained after two passages. The proliferation profile of T-MPCs in our study was significantly different from that of BM-MPCs, with an average population doubling time of 37 hours compared with 58 hours for BM-MPCs. This discrepancy between T-MPCs and BM-MPCs observed in this study is probably in part age-related as it is well documented that BM-MPCs from older donors have a slower proliferation rate at the initial passage up to cell senescence [31,32]. Since the BM-MPCs used here are from old donors whereas the T-MPCs are derived from children, our observation is thus consistent with the previous age-related observations, although intrinsic differences between BM-MPCs and T-MPCs cannot be ruled out.

The CD surface epitope profile of T-MPCs was characterized by immunofluorescence and flow cytometry. Confocal microscopy revealed that both BM-MPCs and T-MPCs expressed CD29, CD44, CD90, and CD105, but hematopoietic surface markers, including CD14, CD34, and CD45, were absent. Furthermore, flow cytometric analyses of T-MPCs confirmed the non-hematopoietic and non-endothelial nature of T-MPCs, based on their lack of expression of CD45 and CD31, respectively. Taken together, these findings showed that T-MPCs share a similar phenotypic profile with BM-MPCs.

The multilineage potential of T-MPCs was shown based on their ability to differentiate into multiple mesenchymal lineages, including fat, bone, and cartilage. Histological analysis clearly showed adipocytes containing lipid droplets, matrix accumulation of sulfated glycosaminoglycans in cell pellets, and areas of mineralization in cultures maintained under adipogenic, chondrogenic, and osteogenic conditions, respectively. However, osteogenically and chondrogenically induced T-MPCs, respectively, expressed bone- and cartilage-associated mRNA transcript markers at a lower level compared with BM-MPCs. A number of groups have assessed the influence of age of MPCs donors, both *in vitro *and *in vivo*, on their differentiation potential. Stenderup and colleagues [33] found no difference in osteogenic and adipogenic differentiation capacity between MPCs from young and old donors. However, an age-related loss of both chondrogenic and osteogenic potential of MPCs has also been reported [31,34]. In our study, we did not observe age-related greater differentiation potential expected for T-MPCs from young donors. We speculate that the lower level of expression of particular markers of differentiation could be the result of prior *in vivo *exposure of T-MPCs to high concentrations of inflammatory cytokines, which are characteristically present in this type of tissue source [35]. In this study, all T-MPCs were obtained from patients undergoing tonsillectomy as a result of chronic tonsillitis. Chronic bacterial infection in the tonsils results in the production of local antibodies, a shift of B- and T-cell ratios, and production of large amounts of pro-inflammatory cytokines, including tumor necrosis factor-alpha (TNF-α). Recently, the addition of TNF-α to human MPCs was shown to suppress the osteogenic medium-induced morphological change from spindle to cuboidal shape and also ALP enhancement. [36]. To address this issue, we are currently analyzing the levels of pro-inflammatory cytokine in T-MPC culture medium. In support of our theory that exposure to pro-inflammatory cytokines suppressed the differentiation capacity of T-MPCs, passage 5 T-MPCs showed significantly enhanced differentiation potential for all three lineages when compared with passage 2 cells of the same patients; this characteristic is contrary to what is observed with BM-MPCs (that is, reduced differentiation capacity is seen with increasing passage number). We speculate that increased passaging not only eliminates tissue-plastic adherent inflammatory cells (monocytes), but also aids in reducing pro-inflammatory cytokine production by cells outside their original environment that might suppress differentiation.

Recently, MPCs have been shown to display immunosuppressive properties both *in vitro *and *in vivo *[10,30,37,38]. MPC inhibition of T-cell proliferation stimulated by allogeneic T cells or non-specific mitogenic stimuli [30,37] affects the expression of activation markers, antigen-specific proliferation (both for naive and memory T cells), cytotoxic T-lymphocyte formation, IFN-γ production by Th1 cells, and interleukin-4 production by Th2 cells. [39,40]. The ability to decrease IFN-γ production, characteristic of the potent suppressive effect of MPCs on T-cell proliferation, is present but less pronounced for T-MPCs compared with BM-MPCs. This finding corroborates with the relatively reduced suppression of IFN-γ, a measure of the proliferative activity of the T-cell population in the assay in the presence of T-MPCs versus BM-MPCs. Interestingly, it was recently reported that IFN-γ level affects MPC function; that is, the dual roles of MPCs as antigen-presenting cells or as immune-suppressor cells depend on IFN-γ levels. Chan and colleagues [41] showed that the antigen-presenting characteristic of MPCs occurs within a narrow window of IFN-γ level (10 U/mL), whereas at 100 U/mL of IFN-γ, MPCs are immunosuppressive. Though speculative, our observations are consistent with the reported low level of IFN-γ (25.6 ± 7.9 IU/mL; *P *< 0.05) produced by tonsillar mononuclear cells compared with the level recorded in the BM sera (41 ± 23 IU/mL; *P *< 0.001), which could explain, in part, the different immunosuppressive characteristics between BM-MPCs and T-MPCs [42-44].

While the immunosuppressive mechanisms of MPCs remain to be clarified, mediation by soluble factors, such as IFN-γ, is strongly suggested. First, it was reported that MPCs immunosuppressed peripheral blood CRTH2^-^CD4^+ ^T cells that produce IFN-γ, but did not affect the proliferation of purified CRTH2^+^CD4^+ ^T cells unable to produce IFN-γ [13,45]. Furthermore, fetal MPCs, normally non-immunosuppressive in an MLR, when exposed to IFN-γ for 7 days, inhibit lymphocyte proliferation at a magnitude similar to that seen with adult MPCs. [38,46,47]. IFN-γ induction of the suppressive effect of MPCs on cell proliferation has been suggested to be related in part to the enhancement of IDO activity [12,13]. IFN-γ/receptor binding leads to subsequent endocytosis and IFN-γ nuclear localization sequence (NLS)-guided binding to the IFN-γ-activated sequence (GAS) response element in the promoter region of IFN-γ-activated genes, such as IDO. [48]. This pathway involving IFN-γ, IFN-γR, and IDO is supported by a study showing complete abrogation of the suppressive potential of MPCs and the ability of IFN-γ to stimulate IDO activity upon treatment with a neutralizing antibody to IFN-γR. [13]. Furthermore, the response to IFN-γ is related and proportional to the level of its receptor; that is, increased IFN-γR expression results in increased IFN-γ signaling and enhanced IDO gene activation. [49]. Our findings are thus consistent with these reports in that the less pronounced immunosuppressive activity of T-MPCs compared with BM-MPCs is associated with a significant fourfold lower expression of IFN-γR1. Also, treatment with neutralizing antibody to IFN-γR1 completely blocked the IFN-γ-stimulated IDO activity in BM-MPCs and T-MPCs. This study suggests, for the first time, a correlation between IFN-γR1 expression level in MPCs from two different tissue sources and their immunosuppressive potency.

The secondary lymphoid organs – lymph nodes, spleen, tonsils, and Peyer's patches – are the sites where immune responses against microbes or antigens are initiated. B lymphocytes that continuously recirculate through the blood and secondary lymphoid organs to encounter antigens or that specifically recognize and respond to antigens located in the tonsils become activated and undergo clonal expansion and somatic hypermutation, leading to differentiation into memory and plasma cells. [50]. The identification of MPCs in tonsils raises interesting questions about their immunosuppressive functions and their effects on B-cell biology in this lymphoid organ. Few studies have addressed the effects of BM-MPCs on B-lymphocyte functions. It has been reported that MPCs inhibit B-lymphocyte proliferation [51-53] and that this activity requires soluble factors [54] such as IFN-γ. [13]. It is noteworthy that B-cell differentiation requires a close association with stromal cells [55,56], and MPCs have been recently shown to give rise to a fully functional population of B-cell supportive fibroblastic reticular cells (FRCs) [57], found associated with the follicular dendritic cells (FDCs) within secondary lymphoid organs, where they play a key role in the initiation and maintenance of efficient immune response. FDCs have been suggested to differentiate from stromal precursors of mesenchymal origin upon interaction with lymphotoxin α1β2 produced by activated B cells. [58]. BM-MPCs have also been shown to acquire a complete FRC phenotype in response to a combination of TNF-α and lymphotoxin-α1β2 [57]. The importance of environmental influences, particularly those related to inflammation, on the immunosuppressive properties of MPCs has been previously stated. [59]. Indeed, the controversy concerning modulation of B-cell functions by MPCs may reflect the differentiation state of MPCs (that is, into FRCs or FDCs) and/or may be the result of microenvironmental signals. Thus, MPC effects on the immune system are modulated not only by cell-to-cell interactions, but also by environmental factors that shape their phenotype and their functions. Accordingly, the functional differences between BM-MPCs and T-MPCs, notably their immunosuppressive potency, most likely reflect their previous environment *in situ*.

MPCs appear to bypass immune rejection, thus making them attractive candidates for allogeneic transplantation. However, *in vivo*, the behavior of the transplanted MPCs is expected to be influenced by their exposure to immune cells and mediators. Another challenging consideration for the clinical use of MPCs and notably T-MPCs, which are isolated from tonsil tissue frequently infected and infused with inflammatory mediators, is to predict how the host tissues will affect the properties of the MPCs. Future studies are necessary to better understand the impact of the inflammatory microenvironment on MPCs for their application in transplantation protocols.

## Conclusion

The issues mentioned above are central to the use of allogeneic MPCs in therapeutic applications. Accordingly, there is recent emphasis on the search for alternative sources of MPCs. Based on the findings reported here, human palatine tonsils could be added as another source of MPCs. More importantly, the presence of MPCs in a secondary lymphoid organ underscores their potential contribution to a specialized microenvironment that supports the initiation and the maintenance of efficient immune responses.

## Abbreviations

AGN = aggrecan; ALP = alkaline phosphatase; BM = bone marrow; BM-MPC = bone marrow-derived mesenchymal progenitor cell; CFU-F = colony-forming unit-fibroblast; COL2 = collagen type II α1; DMEM = Dulbecco's modified Eagle's medium; FBS = fetal bovine serum; FDC = follicular dendritic cell; FITC = fluorescein isothiocyanate; FRC = fibroblastic reticular cell; GAPDH = glyceraldehyde 3-phosphate dehydrogenase; IDO = indoleamine 2,3-dioxygenase; IFN-γ = interferon-gamma; IFN-γR = interferon-gamma receptor; LPL = lipoprotein lipase; MHC = major histocompatibility complex; MLR = mixed lymphocyte reaction; MPC = mesenchymal progenitor cell; NHS = normal human serum; NK = natural killer; OC = osteocalcin; PBMC = peripheral blood mononuclear cell; PBS = phosphate-buffered saline; PE = phycoerythrin; PF = phosphate-buffered saline + 0.1% fetal bovine serum; PHA = phytohemaglutinin; PPARγ = proliferator-activated receptor-gamma; RT-PCR = reverse transcription-polymerase chain reaction; T-MPC = tonsil-derived mesenchymal progenitor cell; TNF-α = tumor necrosis factor-alpha.

## Competing interests

The authors declare that they have no competing interests.

## Authors' contributions

SJ and FD performed experimental work, analyzed and prepared the data and manuscript, and contributed equally to this study. RMS, KG, and LR participated in the cell culture work. DB helped in flow cytometry analysis. DP procured the palatine tonsils for the isolation of mesenchymal progenitor cells. ASJ helped in the analysis of the data. RST participated in the experimental design and data analysis, prepared the manuscript, and supervised the project. All authors read and approved the final manuscript.
